# Rapidly progressive acute necrotizing encephalopathy associated with influenza A in an elderly adult

**DOI:** 10.1002/ams2.611

**Published:** 2020-12-09

**Authors:** Mami Tsubota, Akihiro Kato, Takahiro Goshima, Kazunori Imai, Yota Yamagishi, Asako Matsushima, Hiroshi Sasano, Tomonori Hattori

**Affiliations:** ^1^ Department of Emergency Medicine Nagoya City University Hospital Nagoya Japan

**Keywords:** Acute necrotizing encephalopathy, heatstroke, influenza A, rapidly progressive course

## Abstract

**Background:**

Among the influenza‐associated encephalopathies, acute necrotizing encephalopathy (ANE) has a particularly poor prognosis. While it usually progresses within 48 h, we encountered a rapidly evolving case with the patient falling into coma from lucidity within 10 min.

**Case Presentation:**

A 71‐year‐old man was found unconscious after taking a 10‐min bath and brought to the emergency room. The head computed tomography (HCT) was normal, and he was diagnosed with heatstroke as a complication of influenza A. Despite effective therapy to correct his temperature, his consciousness did not improve, and within 24 h he progressed to multiple organ injury. Repeat HCT and subsequent magnetic resonance imaging revealed irreparably progressed ANE.

**Conclusion:**

To effectively treat ANE, early recognition and diagnosis are critical. Our case suggests that ANE should be considered and added to the differential diagnosis for adult patients with rapid cognitive deterioration.

## Graphical abstract

We encountered a patient with rapidly progressive acute necrotizing encephalopathy associated with influenza A falling into coma from lucidity within 10 min.

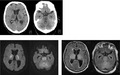

## Introduction

Acute necrotizing encephalopathy (ANE) is one of the influenza‐associated encephalopathies with a rapidly progressing course. Diagnosis depends on characteristic head computed tomography (HCT) and magnetic resonance imaging (MRI) findings, with symmetrical lesions in the thalamus, cerebellum, and brain stem.^1^, ^2^ Many cases have been reported in the pediatric literature, but very few cases in adults. The disease prognosis is extremely poor without any definitive treatment so far. As the only strategy, it is crucial to suspect ANE and provide potential therapies in the early stage.^2^, ^3^ We encountered a case of ANE in an elderly adult who progressed from lucidity to comatose within 10 min.

## Case report

**Fig. 1:**
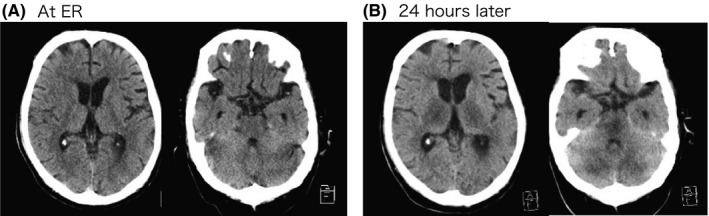
Head computed tomography at (A) emergency room (ER) and (B) 24 h later after the admission. The image at ER had been normal; however, those 24 h later showed low density at the bilateral thalamus, cerebellum, and brain stem.

**Fig. 2:**
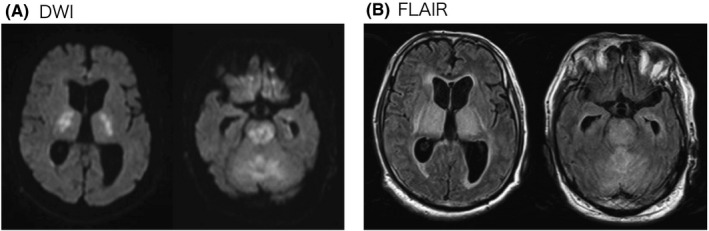
Head magnetic resonance imaging. (A) Diffusion‐weighted imaging (DWI) and (B) fluid‐attenuated inversion recovery (FLAIR) followed by the second head computed tomography. Both images show bilateral symmetric lesion of the thalamus, cerebellum, and brain stem.

The patient was a 71‐year‐old man with a history of diabetes, hypertension, and benign prostatic hyperplasia. Despite malaise for a few days, he was able to talk normally with his family just before taking a hot‐water bath. However, 10 min later, he was found unconscious in the bath and was then brought to the emergency room (ER) in our hospital. At the ER, his vital signs were as follows: respiratory rate, 30 breaths/min; oxygen saturation, 96% on O_2_ 2 L/min; blood pressure, 70/40 mmHg; heart rate, 150 b.p.m.; body temperature (BT), 40.1°C; and level of consciousness (as measured using the Glasgow Coma Scale), E1V1M1. There was no rash or other signs of skin burns. Initial laboratory testing was generally normal except for the slightly elevated C‐reactive protein and creatinine (Table 1). Antigen testing of nasal swab was positive for influenza A. An HCT was normal (Fig. 1A). Taken together, we thought that he had heatstroke due to hot‐water bathing associated with influenza A, and then administered fluid bolus and peramivir (300 mg), and also performed surface cooling. Two hours later, his consciousness slightly recovered to E2V2M3 without limb palsy. Other vital signs also improved, as follows: blood pressure, 110/70 mmHg; heart rate, 120 b.p.m.; and BT, 38.0°C. However, 24 h later, his consciousness remained the same despite his BT returning to normal (36.5°C). The laboratory data were now consistent with multiple organ dysfunction (Table 1). The second HCT showed abnormal hypodensity at both thalamus, cerebellum, and brain stem (Fig. 1B). Furthermore, the subsequently performed head MRI revealed the symmetrical high‐density lesions in the same area, which indicated progressed ANE with severe edema, infarction, and necrosis (Fig. 2A,B). The patient died 30 h later after admission.

**Table 1:** Blood chemistry examination at the presentation to emergency room (ER) and 24 h later

		At ER	24 h later
WBC	/μL	5,300	14,100
Hb	g/dL	15.8	15.2
PLT	/μL	190,000	75,000
PT	%	107.5	(–)
PT‐INR	0.96	(–)	
Fib	mg/dL	338	(–)
FDP	μg/dL	3.9	(–)
D‐Dimer	μg/dL	0.8	(–)
TP	g/dL	8.0	6.1
Alb	g/dL	3.9	2.8
CRP	mg/dL	1.15	3.66
CK	IU/L	50	4,542
AST	IU/L	32	2,159
ALT	IU/L	25	1,197
LDH	IU/L	231	2,230
ALP	IU/L	268	279
Amy	IU/L	65	280
Cre	mg/dL	1.29	1.70
BUN	mg/dL	16.1	35.1
Glucose	mg/dL	182	145
Na	mEq/L	136	138
K	mEq/L	4.0	4.7
Cl	mEq/L	103	103
Ca	mg/dL	9.3	7.9
T‐Bil	mg/dL	0.4	0.7
PCT	ng/mL	0.12	(–)

The data at presentation showed slightly increased CRP and creatinine; however, those obtained at 24 h later showed multiple organ injury involving the liver, kidneys, and muscle.

Alb, albumin; ALP, alkaline phosphatase; ALT, alanine aminotransferase; Amy, amylase; AST, aspartate aminotransferase; BUN, blood urea nitrogen; CK, creatine kinase; Cre, creatinine; CRP, C‐reactive protein; FDP, fibrin degradation products; Fib, fibrinogen; Hb, hemoglobin; LDH, lactate dehydrogenase; PCT, procalcitonin; PLT, platelets; PT, prothrombin time; PT‐INR, prothrombin time‐international normalized ratio; T‐Bil, total bilirubin; TP, total protein; WBC, white blood cells. (–) indicates “not examined.”

## Discussion

In this case, the patient became unconscious during a 10‐min hot‐water bath at home. In the ER he was found to be comatose, hyperthermic, and hypotensive. Despite testing positive for influenza A, influenza‐associated encephalopathy was not suspected given the rapidly progressive course, and the slight improvement of his mental status with the treatment of heatstroke. Because his heat (hot water bath) exposure was for only 10 min, we expected that he would become alert on the next day. However, the patients’ status did not improve, and subsequent HCT and MRI revealed findings consistent with ANE with irreparable brain damage (Figs. 1B and 2).

There are very few reports of ANE associated with influenza in adults. In almost all cases, patients develop deterioration in mentation after presenting with severe influenza infection complicated by other organ dysfunction such as acute lung injury, disseminated intravascular coagulation, or shock,^4^ with the onset of lethargy ensuing several hours to 2 days from the start of neurological symptoms.^2^, ^4^, ^5^ There has never been a case reported in which it progressed to coma this rapidly.

Compared with other acute influenza‐associated encephalopathy in adults, ANE has an extremely poor prognosis.^2^ It was estimated that the mortality rate was about 30% and less than 10% of patients recovered completely while the neurological sequelae were frequent in survivors.^2^, ^3^ The etiology and the pathogenesis of ANE are incompletely understood. The absence of influenza virus in cerebrospinal fluid and autopsy specimens of the central nervous system from the patients has led to the speculation that the triggering inflammatory insult originates outside the central nervous system, as opposed to direct viral invasion of the central nervous system.^3^ It has also been hypothesized that disruption of the blood–brain barrier in the presence of systemic hypercytokinemia associated with the so‐called cytokine storm could be responsible for inducing the necrotic brain lesions that are observed in ANE.^2^, ^3^, ^4^, ^6^, ^7^, ^8^

While there are no proven therapies for ANE associated with influenza, empirical treatment with administration of anti‐influenza drugs and immunomodulatory therapy such as intravenous glucocorticoids (steroid pulse therapy), immunoglobulin, plasmapheresis, and therapeutic hypothermia have been proposed.^2^, ^4^, ^9^ It has been suggested that aggressive intensive care at the early stage of the disease might help prevent the brain stem lesions and improve neurological outcomes.^3^, ^4^, ^9^, ^10^ Once the necrotic lesions extending into the bilateral thalamus and brain stem advance, as in this case, treatment is likely futile.^2^, ^6^

Head MRI might be more effective than CT at detecting early intracranial lesions.^8^ There were reports that the detection at the early stages of ANE using MRI could lead to better outcomes.^4^, ^8^ In this case, if head MRI had been performed earlier, it was possible that ANE might have been detected prior to the development of irreversible intracranial lesion, and might have improved his course. In this case, however, we were not able to suspect encephalopathy associated with influenza, because coma occurring within a relatively short period (10 min) has never been reported.

## Conclusion

We presented a case of ANE associated with influenza A, in which it had taken only 10 min to fall into comatose from consciousness. ANE has an exclusively poor prognosis among patients with encephalopathy associated with influenza. Early detection and early application of intensive care appear to be the only potentially effective therapeutic strategy, and therefore, emergency physicians should be aware that ANE can present with an extremely rapid progressive course.

## Disclosure

Approval of the research protocol: N/A.

Informed Consent: Informed consent was provided by the patient’s family for publication of this case report.

Registry and Registration No. of the study/Trial: N/A.

Animal Studies: N/A.

Conflict of Interest: None declared.
